# Host Plant Apparency and Push–Pull Strategies: A Unified Framework Linking Plant-Mediated Mechanisms for Sustainable Pest Management

**DOI:** 10.3390/insects17060543

**Published:** 2026-05-23

**Authors:** Xinliang Shao, Qin Zhang, Lili Li, Ruxue Tan, Kedong Xu

**Affiliations:** 1 Key Laboratory of Plant Genetics and Molecular Breeding, Zhoukou Normal University, Zhoukou 466001, China; 20211041@zknu.edu.cn (X.S.); lilili@zknu.edu.cn (L.L.);; 2 College of Forestry, Henan Agricultural University, Zhengzhou 450002, China; 3 College of Life Sciences and Agronomy, Zhoukou Normal University, Zhoukou 466001, China; 4 Henan International Joint Laboratory of Translational Biology, Zhoukou Normal University, Zhoukou 466001, China

**Keywords:** host finding, insect herbivory, natural enemy, odor mixing, plant diversity, plant odor

## Abstract

Host-finding behavior of insect herbivores is a key determinant of herbivory intensity in agricultural and forest ecosystems, which often drives excessive pesticide application for pest control. While host plant apparency theory explains herbivore host detection, and push–pull strategies manipulate this behavior, both produce inconsistent results and remain mechanistically disconnected. Existing frameworks like the Resource Concentration Hypothesis focus mainly on host density, ignoring the multidimensional, context-dependent nature of apparency. Here, we synthesize forest and agricultural research to develop the first unified framework linking these two concepts. We show that host plant apparency is not intrinsic but shaped by plant morphology, non-host identity, and spatial arrangement. Push–pull strategies exploit this relativity by redesigning the chemical and physical apparency landscape. We argue that: (1) push–pull system success requires reducing main crop apparency while enhancing trap crop apparency; (2) trap crops may fail when their dual functions, olfactory attraction or physical interception, are misinterpreted, with profound implications for spatial design; and (3) this integration resolves field contradictions by framing them within a common bottom-up mechanism. Our framework provides a generalizable principle for sustainable pest management: effective control depends on understanding what makes host plants apparent to target pests in their specific local environment.

## Simple Summary

Plant-based push–pull pest management strategies show great potential for reducing pesticide reliance, but their inconsistent field performance and lack of a unifying theoretical foundation have hindered widespread adoption. Two key concepts underpin sustainable pest control: host plant apparency (how easily pests detect plants via visual and olfactory cues) and push–pull strategies (using repellent plants to deter pests from main crops and attractive plants to divert them to trap crops). However, these two frameworks have remained largely disconnected at the mechanistic level, leading to contradictory results. This review synthesizes forest and agricultural research to develop the first unified framework linking apparency theory with push–pull practice. We demonstrate that apparency is context-dependent, not intrinsic, and that push–pull systems work by redesigning the “apparency landscape”. We also clarify that trap crop failures often stem from misinterpreting their dual functions (olfactory attraction vs. physical interception). Our framework provides a generalizable principle for evidence-based sustainable pest management.

## 1. Introduction

In multitrophic interactions, the host-finding behavior of insect herbivores is a critical process that dictates herbivory patterns and, consequently, plant fitness and ecosystem dynamics [1,2]. Insect herbivores typically employ a hierarchical host-search strategy, progressing from landscape-scale orientation to individual plant-level discrimination, with the initial search phase largely determined by their proximity to host resources [3,4,5]. Plant-related factors, including individual morphology (e.g., size, height), species diversity, and spatial configuration, directly shape pest distribution and population density by modulating how herbivores locate hosts, often functioning independently of top-down regulation by natural enemies [5,6,7,8,9,10,11]. For example, increased plant diversity can reduce pest infestation on focal host plants due to the disruptive cues created by non-host neighbors, which hinder host finding by insect herbivores [12,13,14]. However, predicting the magnitude and direction of plant diversity effects on insect herbivory remains challenging, as it may also enhance herbivory or have no effect [15,16,17,18].

To address this complexity, the concept of host plant apparency, defined as the detectability and accessibility of host plants to insect herbivores, has emerged as a robust predictor of herbivory patterns across diverse plant communities [10,19,20,21,22]. Higher apparency, often linked to plant height, structural prominence, and edge location, correlates with increased herbivore pressure. For instance, in sapling communities, the density of leaf miners significantly increased with the apparency of oak saplings (*Quercus robur*) [10]. Martini et al. (2021) suggested that host plant apparency may be the most important driver of insect herbivory on tree seedlings [23]. In pure stands of maritime pine (*Pinus pinaster*), taller pine trees and those located near stand boundaries are more vulnerable to infestations by pine processionary moth (*Thaumetopoea pityocampa*) [24]. In mixed stands, taller non-host birch trees (*Betula pendula*) planted within and around pine stands (*P. pinaster*) significantly reduced the attack probability of *T. pityocampa* on host pine trees [25], but the protective effect of birch faded with time and disappeared once the pines grew taller than the birches [26]. Similarly, infestations of the bark beetle (*Pityogenes chalcographus*) were found to be lower on all host trees of six conifer species that were less apparent, located deeper within forest stands and hidden among other trees [27]. Other studies have also indicated that insect herbivory on *Quercus robur* [28], *Anadenanthera macrocarpa* [29], and *Populus laurifolia* [30] increased with the height of the trees. While numerous studies have established a positive correlation between host plant apparency and insect herbivory [10,21,30], inconsistent findings have emerged. For example, Zhang et al. (2023) found that plant apparency had no effect on insect herbivory in a tropical rainforest in southern China [31], highlighting gaps in understanding how context, such as non-host species identity and spatial arrangement, modulates apparency.

Parallel to ecological research on apparency, plant-based push–pull strategies represent a promising applied approach to manipulate herbivore host finding for pest management. These strategies use a “push” component, often companion plants that release repellent volatile organic compounds (odors), to deter pests from the focal crop/plant and a “pull” component, such as trap crops/plants producing attractive odors, to divert pests away, and have been employed to reduce damage to focal crops/plants in agriculture and forestry [32,33,34,35,36,37,38]. Notably, Cook et al. (2007) first formally defined and validated this strategy, providing a foundational framework for its application [36]. In agriculture, particularly, using non-host plants to deter insect pests and protect host plants is a well-regarded strategy for sustainable pest management [2,36,39]. However, non-host companion plants may lead to reductions [40,41], increases [42,43], or no change in insect herbivory on focal host plants [43]. While successful implementation of plant-based push–pull strategies in real-world practice has been documented in small-scale cereal farming systems across sub-Saharan African countries (e.g., Kenya, Uganda, Tanzania) [44,45,46], their replication across other crops and scaling up to intensive agricultural systems remain limited. These inconsistent results, low success rates in real-world implementations [36,39], and thus limited practical scalability are exacerbated by the perceived cheapness, ease of use, and reliability of synthetic insecticides, and indicate unresolved mechanistic questions about how plant factors drive push–pull efficacy. Recent studies, such as that of Magnin et al. (2025) have begun to address these gaps by dissecting the roles of visual, physical, and chemical disruption in push components for oilseed rape pest management [47].

We argue that the ecological concept of host plant apparency provides a powerful theoretical lens through which to evaluate and refine push–pull strategies. Specifically, while apparency theory helps explain why pests find certain plants more easily (via visual and chemical cues), push–pull strategies represent a practical attempt to manipulate herbivore host-finding behavior by exploiting the relativity of host plant apparency. However, existing reviews have largely treated apparency theory and push–pull strategies as separate domains, with the former often limited to visual cues and the latter to olfactory cues [36,48]. Consequently, insights from forest ecology on apparency have rarely been applied to the design and assessment of agricultural push–pull systems. This gap may underlie our limited ability to predict the efficacy of plant-based pest management. While cross-ecosystem generalization requires caution (given habitat-specific pest behaviors), core host-location mechanisms (e.g., response to visual/chemical cues) are conserved, making such integration valuable. For example, forest studies on associational resistance [25,26] can provide valuable insights for agricultural border trap crop design [44,49], while agricultural research on odor-mediated push effects [37,41,50] can help elucidate chemical apparency in forest systems [12,27]. Additionally, insights from maize-based push–pull systems into trap crop functionality [36,45] can complement findings from vegetable system studies [51,52] to enhance generalizability.

In this review, we therefore integrate the concept of host plant apparency with the practice of push–pull strategies under a common, bottom-up framework focused on plant-mediated mechanisms. We synthesize literature from both forest and agricultural ecosystems to: (1) elucidate how plant factors shape host apparency to insect herbivores, and (2) use these insights to critically examine the functioning and limitations of the “push” and “pull” components in pest management systems, and (3) develop generalizable, evidence-based design principles for apparency-driven push–pull strategies. Notably, tritrophic interactions (including effects on natural enemies) are excluded from this review to maintain focus on plant-mediated mechanisms, though we acknowledge their importance in broader pest management. By bridging this theoretical-applied divide, we aim to identify key knowledge gaps and propose more mechanistic and generalizable approaches for developing sustainable pest management strategies.

## 2. Overview of Insect Herbivore Host Finding

Finding suitable host plants for feeding and oviposition is a fundamental determinant of fitness in herbivorous insects [53,54]. Understanding this host-finding process, which is described as a hierarchical sequence of orientation, landing, and acceptance [3,4,55], is essential for deciphering plant–herbivore interactions and developing sustainable pest management strategies [54]. Insects primarily use olfaction and vision for orientation during host finding [55], with most insect herbivores relying primarily on olfactory cues to locate hosts at long distances, supplemented by visual cues for short-range orientation [3,5,55,56]. Visser (1988) and Finch & Collier (2000) further detailed this process, emphasizing that host finding involves sequential responses to plant cues across spatial scales [3,4].

**Figure 1:**
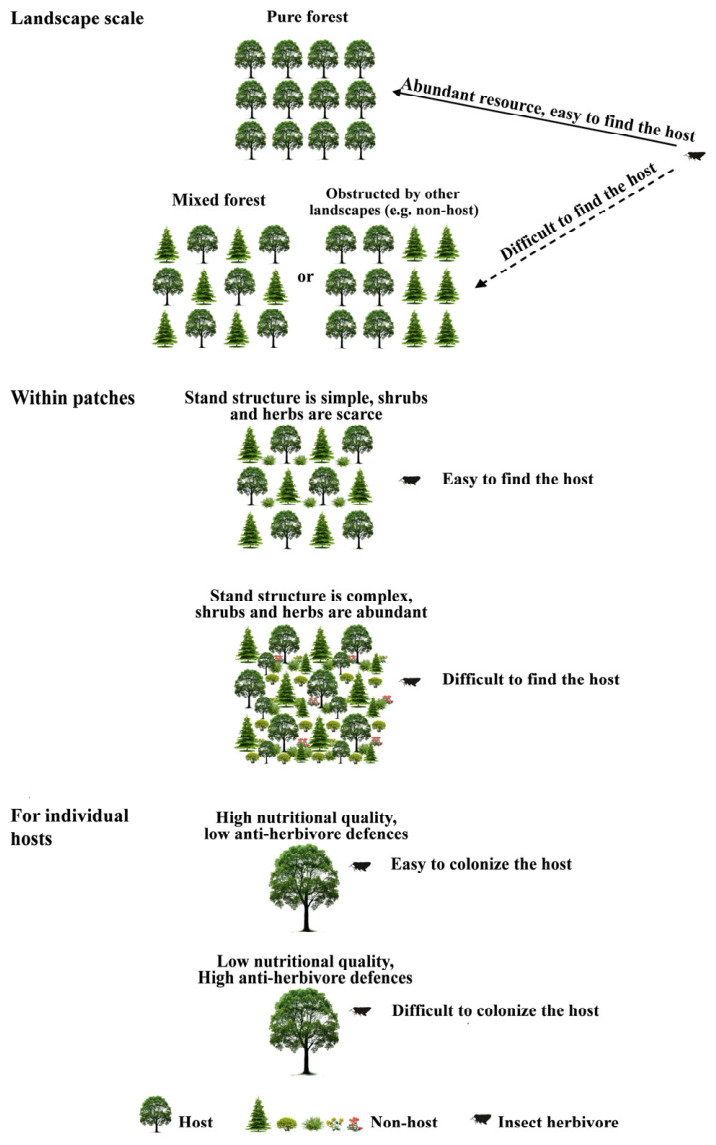
Conceptual diagram illustrating the main factors influencing insect herbivore host finding across three ecological scales: landscape scale, within-patch scale, and individual-host scale.

Insect herbivores’ host finding is mediated by multiple plant factors acting across spatial scales (landscape, within-patch, individual plant; see Figure 1) [8,10,21,57]. To illustrate this general multi-scale framework, we draw on examples from forest ecosystems. At the landscape scale, insect herbivores are more likely to locate patches with abundant host plants, which exhibit high chemical and physical apparency [27,58,59,60,61,62]. These cues help insects navigate large distances and prioritize resource-rich areas at a broad scale [54,63,64]. Within patches/stands, non-host plant abundance, species diversity, and stand structural complexity (e.g., stratification, spatial arrangement, age heterogeneity) modulate host detectability [63,64,65]. Higher non-host diversity or complex stand architectures can reduce host apparency by diluting chemical signals or creating physical barriers, making host location more challenging [10,12,23,60]. At the individual plant scale, host plants with high nutritional quality and low anti-herbivore defensive traits (e.g., fewer secondary metabolites) are often more attractive and prone to colonization by insect herbivores [66,67,68] (Figure 1). This hierarchical progression from landscape-scale orientation to individual plant-level discrimination highlights the need to integrate multi-scale plant factors when studying plant-mediated host-finding behavior and designing pest management strategies.

## 3. “Host Plant Apparency” as a Relative and Multidimensional Concept

**Figure 2:**
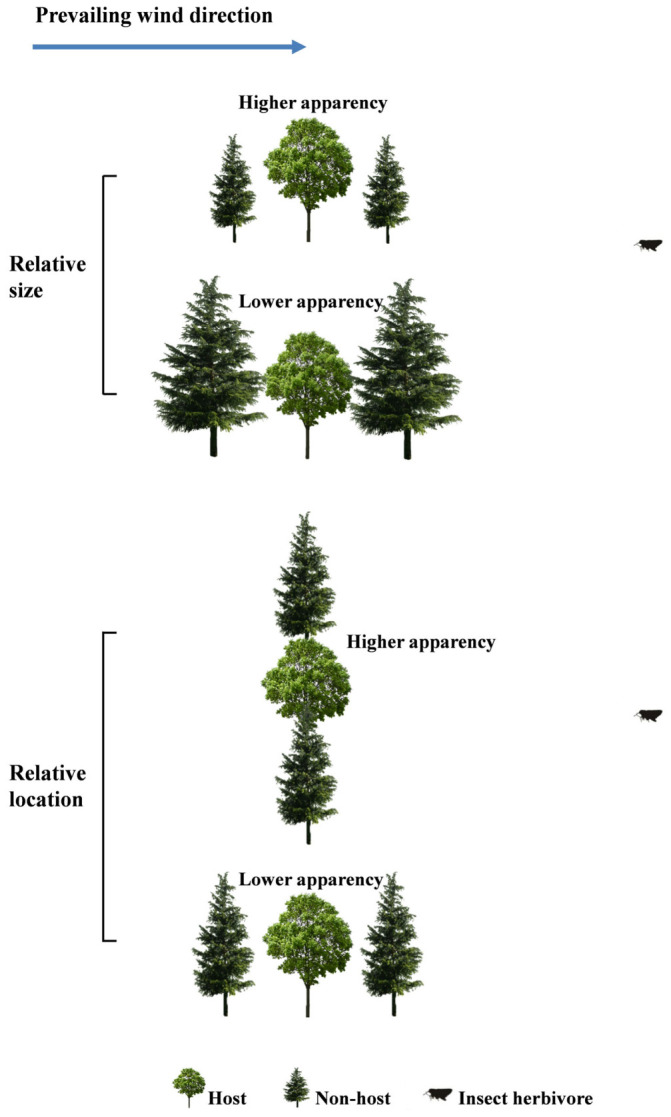
Schematic illustration of host plant apparency for an insect herbivore. For a given herbivore species and specific host/non-host plant pairing, host plant apparency is a relative trait that depends primarily on the relative size (height, canopy dimensions) and spatial arrangement (proximity, wind-directional positioning) of host and non-host plants. The figure illustrates how upwind/downwind placement of non-host plants maximizes both physical obstruction and odor blending, thereby minimizing host apparency, whereas crosswind placement has a weaker masking effect.

The concept of host plant apparency represents a significant advance over earlier frameworks such as the Resource Concentration Hypothesis (RCH), which posits that herbivores are more likely to find and colonize hosts in high-density patches [61,62]. While the RCH provides a useful first approximation, it fails to explain why herbivory often varies dramatically among individual plants within the same density patch, or why low-density host patches can sometimes experience higher herbivory than high-density patches [10,23]. These inconsistencies arise because the RCH treats host detection as a simple function of resource abundance, ignoring the critical roles of relative plant size, non-host interference, and spatial configuration in shaping herbivore perception of the landscape. The concept of host plant apparency is inherently relative, as it describes a plant’s detectability to insect herbivores within its surrounding environment. While often simplified as plant height or the height difference between focal plants and their neighbors [10,23,31,69], plant apparency is not solely determined by absolute size: a smaller host plant may exhibit greater apparency than a larger counterpart if it is positioned to be more conspicuous (visually or chemically) within its microenvironment (Figure 2). We propose that apparency is best understood as a multidimensional property with both physical and chemical components.

Physical apparency relates to a plant’s visual and structural conspicuousness. This relativity arises from two key dimensions: relative size (e.g., height, canopy volume) and the spatial arrangement of host and non-host plants [50,70,71]. Critically, the locations of non-host plants relative to the host can significantly influence herbivore host-finding success by affecting both the physical and chemical apparency of the focal host plant [5,50,65,71]. For example, a host-finding insect herbivore may have more difficulty locating its host when non-host plants are positioned both upwind and downwind compared to a crosswind position (Figure 2). Physically, non-host plants located upwind and downwind could create structural barriers that impede herbivore movement or visual detection (Figure 2). Chemical apparency refers to the detectability of a plant’s volatile organic compounds. This is not an intrinsic property of the host alone but is profoundly affected by neighboring plants. The degree of odor mixing between host and non-host plants is significantly higher when non-host plants are positioned upwind/downwind rather than in crosswind positions, which can interfere with the insect herbivore’s ability to discriminate and track host odor plumes [5,50,70,71] (Figure 2). Non-host plant odors effectively reduce host chemical apparency only when two conditions are met: (1) the adjacent non-host plant odor is less attractive to the insect herbivore than the host odor, and (2) the degree of odor mixing between host and non-host plants reaches a certain level that disrupts the insect’s recognition of host odors [5]. This masking effect is particularly relevant in agricultural settings, where non-host odors can reduce host detectability even if they are not strongly repellent [47].

Spatial proximity between host and non-host plants further modulates these effects: tighter spacing enhances both odor blending and physical obstruction, amplifying the reduction in host apparency [50,70]. Strategies such as intercropping, border planting, or companion planting can effectively hide host plants in agricultural environments by leveraging both physical and chemical disruption [36,47,51]. Thus, host plant apparency is best understood as a composite property, emerging from the interaction between plant morphology, neighbor identity, and spatial arrangement, which collectively determine how easily herbivores can locate hosts within complex plant communities (Figure 1 and Figure 2).

## 4. Conditions for Host Plant Apparency to Influence Herbivory

The predictive validity of host plant apparency depends on both intraspecific comparison and herbivore source differentiation. First, host plant apparency should be assessed through intraspecific comparisons (i.e., within the same plant species), as interspecific evaluations may confound its relationship with herbivory patterns. Interspecific variation in inherent odor emission, palatability and defensive traits means that larger and highly apparent plant species may experience less herbivory if they are less attractive, and chemically or physically defended, while smaller, less apparent but more attractive and palatable species could suffer greater damage. While canopy height serves as a reliable proxy for apparency in intraspecific comparisons, its application across species may yield misleading conclusions (e.g., [31]), since herbivore preferences shaped by plant odors and nutritional quality often supersede structural apparency in diverse plant communities [3,55,56,69].

**Figure 3:**
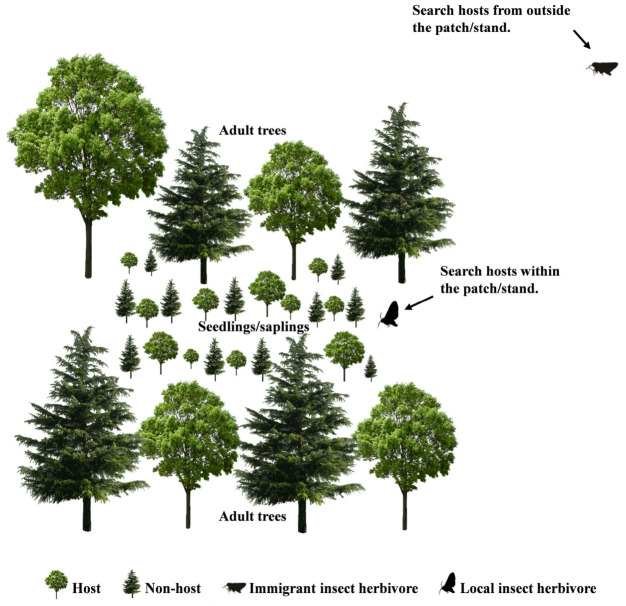
Influence of herbivore origin on the role of host plant apparency in regulating insect herbivory. For immigrant herbivores, the apparency of adult host trees significantly impacts their host-finding success and subsequent herbivory on these trees. In contrast, local herbivores are influenced by the apparency of both adult hosts and seedling/saplings, as their fine-scale navigation within the patch makes both life stages relevant to host selection and subsequent herbivory.

Furthermore, the strength and direction of apparency effects are mediated by herbivore population origin, showing significantly different patterns between locally adapted residents and immigrant populations. In forest systems, for instance, immigrant herbivores such as moths or beetles that disperse into a stand rely strongly on the apparency of adult host trees for long-distance orientation, as tall, conspicuous plants provide enhanced visual and chemical cues (Figure 3). This is particularly evident in edge effects, where the higher apparency of trees at stand boundaries leads to consistently greater infestation pressure compared to interior trees [24,27]. In contrast, seedling/sapling apparency likely has minimal influence on immigrant herbivores, whose dispersal strategies primarily utilize landscape-scale cues (Figure 3).

Conversely, for resident insect herbivores within the stand (e.g., short-range flyers), both adult and juvenile host apparency significantly influence host finding. Studies show that resident herbivores exhibit a consistent preference for apparent hosts, whether seedlings/saplings [23] or adults [19], driven by their reliance on fine-scale visual and chemical cues during local foraging (Figure 3). These context-dependent effects underscore the critical need to differentiate between resident and immigrant herbivore populations when investigating how host plant apparency regulates insect herbivory. In agricultural systems, however, a critical distinction arises. Due to the typically much smaller size of crop fields compared to forest stands, most insect pests are effectively “immigrants”, arriving from outside the field boundaries. In this context, the apparency of both taller/larger plants within the field and of plants at the field edges becomes a major driver of host-finding success for these pests [36]. This apparency landscape is precisely what push–pull strategies aim to manage.

## 5. Plant-Based Push–Pull Strategy: Managing the Apparency Landscape for Crop Protection

Although pesticides offer effective short-term pest control, their extensive use leads to environmental contamination, health risks, and rapid evolution of insect resistance (typically within 60–78 generations) [72,73]. As an ecological alternative, the plant-based push–pull strategy has been adopted in agriculture and forestry to protect target crops/plants [32,33,34,35,36,37,38,39]. It is important to note that while this review focuses on plant-based mechanisms, forestry applications often incorporate synthetic chemicals (e.g., verbenone as a “push”, ethanol lures as a “pull”), with variable reported efficacy [35,74,75,76,77]. To date, well-documented success for plant-based push–pull systems remains largely confined to smallholder agriculture [44,45,46], with limited comparable evidence from forest ecosystems. Despite implementation differences, the core behavioral and ecological principles are aligned across systems. Therefore, the following discussion primarily draws on agricultural research to explicate the mechanisms of plant-based repellents and attractants.

### 5.1. Core Components of Push–Pull Systems

**Figure 4:**
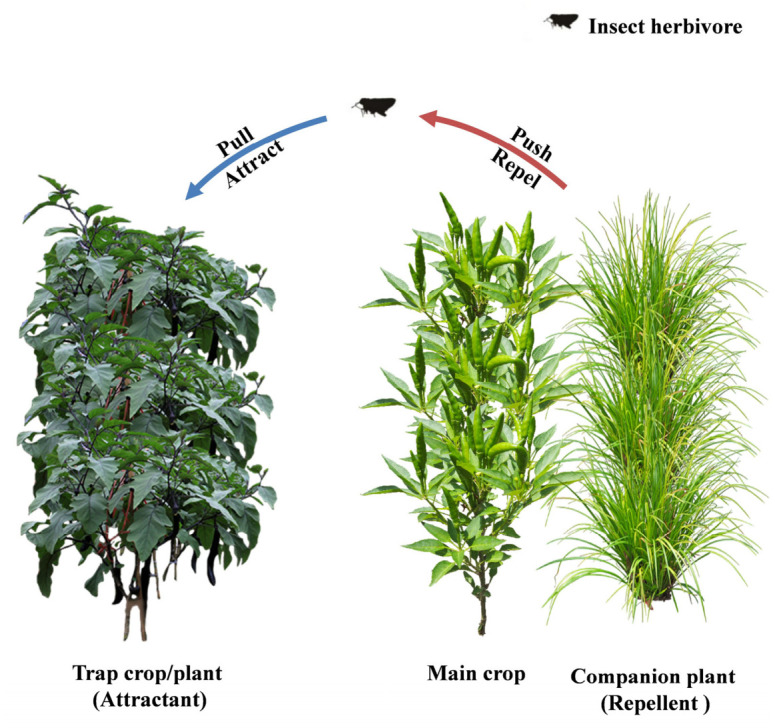
Schematic diagram of the core components and functioning of a plant-based push–pull strategy in agricultural systems.

An optimal push–pull strategy integrates two components (Figure 4): (1) Push elements, such as a companion crop that is repellent or less attractive than the main crop, which deter pests by reducing host plant apparency via physical obstruction, odor masking, or repellent odor emission. For example, Magnin et al. (2025) demonstrated that intercropping oilseed rape (*Brassica napus*) with faba beans (*Vicia faba*) reduced pest infestation on oilseed rape via visual and physical disruption [47], while *Desmodium uncinatum* releases repellent odors to deter stemborers in maize [44]. (2) Pull elements, such as highly attractive trap plants, which further diminish pest pressure by offering a more apparent and attractive alternative, luring herbivores away from the main crop. Ideal trap crops should fulfill a dual function: serving as highly attractive sinks for herbivores while simultaneously suppressing pest fitness through antibiosis or other inhibitory mechanisms (i.e., “dead-end” trap crops) [78,79]. For instance, yellow rocket (*Barbarea vulgaris*) acts as a dead-end trap crop for diamondback moth (*Plutella xylostella*), as females preferentially oviposit on it, but larvae cannot survive due to triterpenoid saponins [78,80]. Similarly, *Raphanus sativus* reduces pollen beetle (*Meligethes aeneus*) survival by 35% while attracting oviposition [79]. Napier grass (*Pennisetum purpureum*) and Vetiver grass (*Vetiveria zizanioides*) also function as effective dead-end trap crops for stemborer pests (*Busseola fusca* and *Chilo partellus*) in Africa [44,49,81,82].

This dual mechanism manages the “apparency landscape” to disrupt pest orientation and redistribute herbivore populations, thereby offering protection to the primary crops (Figure 4). Notably, while natural enemies also enhance push–pull efficacy by using plant odors to locate herbivores and by benefiting from shelter or alternative resources provided by the system [36,39], this tritrophic interaction is not the focus of the current review.

### 5.2. Challenges and Mechanistic Uncertainties

The deployment of trap crops/plants entails an inherent ecological risk: their attractant properties may concentrate herbivore populations within the field or at its margins, potentially increasing pest encounter rates with adjacent main crops. This could compromise the “push” function of companion plants while exacerbating pest pressure on target crops [5]. Elevated pest densities on trap crops may lead to spillover colonization of target crops, a phenomenon mirroring herbivore dispersal in forest ecosystems [27].

**Figure 5:**
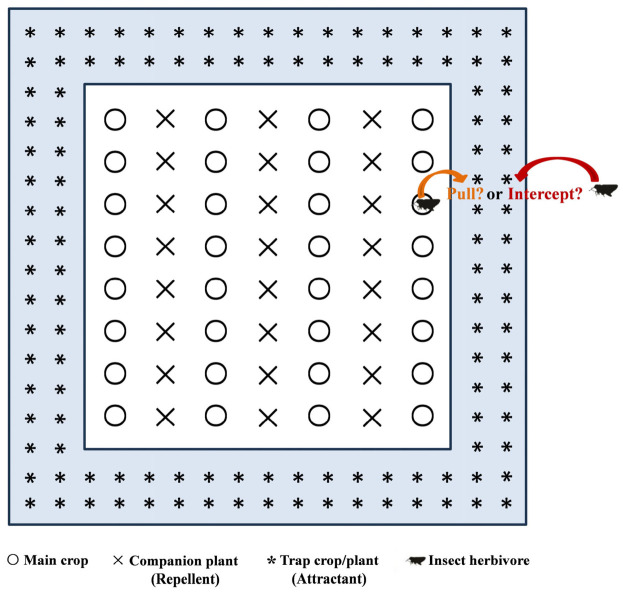
Schematic illustration of successful push–pull strategy implementations, depicting how the trap crop/plant influences insect herbivore host finding. The observed reduction in main crop pest load remains ambiguous, as it could stem from either “pull” effects (attracting pests away from plot centers, orange arrow) or physical barrier effects (intercepting externally derived pests, red arrow). This key distinction, which is critical to understanding trap plant functionality, has yet to be conclusively resolved in existing research. To resolve this ambiguity, targeted experiments are proposed comparing pest colonization patterns in plots with peripheral versus internal trap crop placement, combined with pest origin marking to quantify interception versus attraction.

A key challenge persists in identifying optimal trap crops/plants that simultaneously satisfy two critical criteria: superior emission of pest-attractive odors compared to the main crop, and significant suppression of pest fitness parameters [78,79]. Even in successful cases, the underlying mechanisms of pest load reduction remain unclear: do trap crops primarily function as olfactory attractants that divert pests from the main crop (pull effect), or do they serve as physical barriers that intercept dispersing pests? Analyses of successful trap crop systems consistently identify two key features: (1) implementation at relatively small spatial scales (typically < 30 × 30 m) [36,45,49,51], and (2) strategic perimeter planting of highly attractive trap species (e.g., Napier grass (*Pennisetum purpureum*) or Sudan grass (*Sorghum drummondii*)) encircling the main crop [36,44] (Figure 5). This spatial configuration leaves uncertainty about whether pest reduction stems from pests being drawn away from the plot interior towards the peripheral trap crops (attraction-based diversion), or from the physical interception of pests immigrating from outside. This distinction parallels observations in forest edge effect dynamics, where visually conspicuous perimeter vegetation similarly modulates the behavior of immigrating pest populations [24,27]. Potting et al. (2005) demonstrated through simulation modeling that the efficacy of a trap crop is co-determined by the specific host-finding behavior of the target herbivore (including colonization pattern, movement speed, and sensory mode) and the spatial configuration of the trap crop (e.g., border, intercropped rows, or patches) [83]. A recent simulation modeling study of push–pull management for the ambrosia beetle (*Euwallacea fornicatus*) in avocado orchards reinforces this mechanistic uncertainty. The model demonstrated significantly higher trap capture rates for peripherally placed traps intercepting externally originating beetles compared to those capturing internally emerging populations [84].

Consequently, modifications to either main crop plot dimensions or the spatial arrangement of surrounding trap plants may compromise the “pull” effect efficacy. Such alterations could not only reduce the overall effectiveness of the strategy but, in some cases, may produce unintended counterproductive outcomes. To resolve this longstanding ambiguity, we propose three targeted experimental approaches to quantify the relative contributions of olfactory attraction and physical interception in trap crop systems. First, large-plot comparative experiments can establish three matched treatments in identical main crop plots: continuous perimeter trap crops testing both mechanisms simultaneously, trap crops placed exclusively in the plot interior isolating the olfactory pull effect as internal traps cannot intercept immigrating pests, and no-trap crop controls. Comparing pest pressure on main crops across treatments will allow direct quantification of each mechanism’s contribution. Second, wind tunnel assays can conduct controlled laboratory experiments using artificial plant structures to isolate physical and chemical cues, testing pest responses to trap crop odors alone for olfactory attraction, inert physical structures matching trap crop architecture for physical interception, and combined odors and physical structures for the full effect. Third, mark–release–recapture studies can track the movement paths of individually marked pests in field plots with perimeter trap crops, where pests that land directly on trap crops from outside the plot are likely intercepted, while those that first enter the main crop then move to trap crops are likely attracted by olfactory cues.

This mechanistic ambiguity and context-dependent efficacy directly explain why push–pull strategies often lack broad transferability across cropping systems and regions. Rooted in the failure to account for the multidimensional and context-dependent nature of host plant apparency, such systems are often designed based on empirical trial rather than mechanistic principles, which further limits their generalization beyond narrow conditions. As highlighted by Eigenbrode et al. (2016) and Legaspi et al. (2023), the successful stem borer push–pull programs in sub-Saharan Africa show limited transferability to other agricultural systems [85], especially intensive farming systems [39]. While plant-based push–pull strategies have demonstrated potential in various agricultural systems, their efficacy is often context-specific. For instance, the Vegetable Integrated Push–Pull (VIPP) system employs *Desmodium uncinatum* as a repellent and *Brachiaria* grass as an attractant, effectively suppressing leafminers, whiteflies, and fruit flies on tomatoes and kales [51,52]. However, this same system shows species-specific limitations, failing to exert significant control over generalist pests such as thrips and bollworms on tomatoes, or cabbage loopers on kales [51,52]. This discrepancy underscores that push–pull efficacy is not universally applicable, revealing substantial gaps between experimental findings and real-world implementation. Field studies have further documented inconsistent performance and technical constraints in pest management. For instance, an integrated push–pull system employing tomato (repellent) and winter rape (attractant) failed to reduce *Phyllotreta striolata* infestation on radish, even when supplemented with allyl isothiocyanate (AITC), a host plant volatile attractant, due to the striped flea beetle’s strong host fidelity and the trap crop’s insufficient attraction [86]. For maize systems, similar context dependency emerges: a multi-landscape study in Ethiopia found that the classic maize–Desmodium–Napier grass push–pull system reduced stemborer (*Busseola fusca*) infestation only in landscapes of intermediate complexity, while proving ineffective in simple (maize-dominated) or complex (perennial vegetation-rich) landscapes [87]. This landscape-mediated variability highlights the context-dependent functionality of both “push” and “pull” components. Collectively, these challenges underscore that resolving the mechanistic uncertainty of trap crop function and addressing the context-dependent constraints are indispensable prerequisites for developing robust, scalable push–pull systems that transcend narrow environmental and landscape limitations.

### 5.3. The Critical Role of “Push” Components

Recent research highlights two critical considerations: while these strategies can face limitations in intensive agricultural systems [39], their effectiveness in certain contexts may depend predominantly on the “push” component [48,88]. Theoretical considerations suggest that the “push” component plays a particularly critical role for two reasons. First, from a mechanistic perspective, reducing the apparency of the main crop is a more direct and reliable intervention than attempting to divert pests with a more apparent alternative, given the inherent difficulty of identifying trap plants that are consistently more attractive across variable environmental conditions. Second, empirical evidence indicates that push plants can effectively disrupt herbivore host finding through multiple complementary mechanisms: emitting repellent odors [44,46], masking host attractant odors [50], or forming physical and visual barriers [47]. This multi-modal disruption makes the push component more robust to environmental variation compared to the pull component, which relies on the more variable process of olfactory attraction.

Importantly, this emphasis on the push component does not diminish the value of pull components in successful push–pull systems; rather, it clarifies their complementary roles. The most effective push–pull systems worldwide, including the iconic maize–Desmodium–Napier grass system in East Africa [44,45] and the Vegetable Integrated Push–Pull (VIPP) system [51,52], achieve high efficacy through the synergistic interaction of both components. The push component provides foundational protection by reducing main crop apparency through both chemical (repellent odor emission, odor masking) and physical (canopy shading, structural obstruction) mechanisms, preventing most pests from detecting the main crop in the first place. The pull component then acts as a secondary defense, diverting the small proportion of pests that still manage to locate the main crop patch. In the absence of an effective push component, pull components alone often fail because they cannot prevent large numbers of pests from detecting and colonizing the main crop. Conversely, while push components alone can provide significant protection, adding a well-designed pull component can further reduce pest pressure, particularly in high-infestation environments.

An alternative approach to selecting trap plants can be derived from the “plant apparency” hypothesis: even crops with lower intrinsic attractiveness than the main crop may function as effective traps if their larger size, which is often associated with higher odor emission, preferentially attracts ovipositing adult females [48]. However, this hypothesis should not be misinterpreted to imply that any large plant will suffice. The effectiveness of a trap crop remains contingent upon a complex interplay of factors, including its specific chemical attractiveness (volatile profile), its ability to suppress pest fitness (e.g., as a ‘dead-end’ trap), and crucially, its phenological synchrony with the pest—ensuring that the trap is attractive and present during key pest activity periods. Consequently, while plant size and associated apparency are important considerations, they are not standalone criteria for trap crop selection. Furthermore, the efficacy of this size-dependent mechanism may itself vary across different plant growth stages and seasons, necessitating further field validation to assess its practical applicability.

The efficacy of push components stems from their capacity to diminish host plant apparency through distinct yet complementary mechanisms. As elucidated in previous sections, non-host plants can reduce detectability by masking host odors/emitting directly repellent volatiles (chemical apparency) or creating physical/visual barriers (physical apparency) [4,36,47]. Therefore, selecting an effective push plant requires more than an arbitrary choice; it necessitates identifying species that possess specific bioactive traits, such as particular repellent volatile profiles or growth architectures, capable of actively disrupting herbivore host-finding behavior [47,50].

### 5.4. A Unified Framework for Apparency-Based Pest Management

By framing push–pull strategies through the lens of host plant apparency, we can move beyond context-dependent case studies towards generalizable design principles. The success of a push–pull system hinges on deliberately redesigning the chemical and physical apparency landscape. This involves two concurrent goals: first, minimize the apparency of the main crop. This is achieved by selecting non-host plants that are less attractive than the main crop and capable of high odor mixing, and arranging them (e.g., upwind and downwind positions, tight spacing) to maximize both chemical and physical disruption. Second, maximize the apparency of the trap crop (Pull) while ensuring it functions as a dead-end sink. This is achieved by selecting plants that are intrinsically more attractive than the main crop and structurally apparent, and arranging them (e.g., perimeter planting) to intercept incoming pests effectively. Crucially, to guarantee that increased apparency translates into effective pest control, trap crops must also suppress pest fitness through antibiosis or other inhibitory mechanisms (i.e., function as “dead-end” traps). Without this suppressive function, their effective management would rely on additional interventions, such as targeted insecticide application or timely removal of infested plants, which reintroduce pesticide use and increase labor costs. Otherwise, they risk becoming “source” habitats that amplify local pest populations and exacerbate damage to the main crop. This framework resolves apparent contradictions in both fields. The positive correlation between apparency and herbivory explains why a highly apparent trap crop can be effective. Conversely, the failure of some push–pull systems can be understood as a failure to adequately reduce the apparency of the main crop or to create a sufficiently more apparent and fitness-suppressing alternative under specific landscape and pest origin conditions.

To facilitate the translation of this framework into field application, we provide five evidence-based, actionable recommendations for farmers and forest managers. First, characterize the biology of the target pest to determine whether the primary population consists of immigrants dispersing from outside the management unit and relying on long-distance cues, or residents foraging locally and relying on fine-scale cues. Immigrant pests are most effectively controlled by perimeter trap crops for physical interception and upwind push plants, whereas resident pests respond better to intercropped push plants and distributed pull components. Second, optimize push plant placement for maximum disruption by both positioning repellent companion plants upwind and downwind of the main crop relative to the prevailing wind direction and using tight inter-row spacing, thereby enhancing odor mixing and chemical masking while also increasing physical obstruction and visual disruption of main crop apparency. Third, design trap crops for dual efficacy by selecting species that are both more apparent than the main crop (e.g., greater height and higher odor emission rates) and function as dead-end sinks where larvae cannot survive or develop normally, and by planting trap crops in continuous, unbroken perimeters to maximize interception of immigrating pests. Fourth, ensure phenological synchrony by aligning the planting dates of push and pull plants with the main crop so that all components are at their optimal growth stage, and thus optimal apparency, during key pest activity periods. Fifth, adopt an adaptive management approach by starting with small-scale pilot plots to test different plant species, spacings, and configurations, monitoring pest populations and crop damage regularly, and adjusting the system based on local responses before scaling up to larger areas.

## 6. Conclusions and Future Directions

Host plant apparency, shaped by plant morphological traits (e.g., height, architecture), chemical traits (e.g., odor emission), and community-level interactions (e.g., plant diversity, spatial configuration), serves as a foundational ecological concept for understanding how plant factors shape herbivore host finding. Push–pull strategies represent a translational application of this concept, specifically designed to manage the apparency landscape and thereby manipulate herbivore host-finding behavior within a given context. Our synthesis demonstrates that host plant apparency is not an absolute attribute but a context-dependent property, and it is precisely this relativity that provides the leverage for push–pull interventions. The strategy’s success hinges on deliberately redesigning the chemical and physical landscape: reducing the apparency of the main crop (push) while offering a more apparent and attractive alternative (pull). Thus, while apparency theory offers a powerful lens for understanding why pests locate certain plants, the push–pull approach provides a practical framework for how to intervene in this host-finding process for pest suppression. The apparent tension between apparency as a general predictor and its context-dependency is, in fact, central to its utility: effective push–pull design requires a nuanced understanding of how specific plant factors and spatial configurations jointly determine the apparency landscape in a given farming or forest context.

Key priorities for future research should focus on: (1) Elucidating the impact of non-host plants on chemical apparency through experiments that manipulate wind patterns and spatial configurations. The goal is to quantify effects on odor plume structure and herbivore foraging to guide the selection of optimal non-host species. (2) Distinguishing the mechanistic contributions of “pull” (olfactory attraction) and barrier effects (physical interception) in trap cropping. This requires targeted experiments, such as large-plot comparisons of peripherally versus internally placed traps, to quantify their respective roles in pest reduction. (3) Most critically, advancing beyond context-specific case studies to develop generalizable design principles for designing and testing scalable push–pull configurations compatible with intensive farming systems. Promising approaches include strategically orienting strip intercropping (e.g., orienting strips perpendicular to local prevailing wind directions, a design that directly tests the principles of odor mixing and apparency reduction illustrated in Figure 2), as it maximizes both the physical barrier function and “push” effects of non-host plants, alongside multi-edge non-host borders for efficient interception of immigrating pests.

In summary, integrating theoretical insights from host plant apparency with the practical implementation of push–pull strategies provides a coherent pathway to advance sustainable pest management. This synthesis not only helps resolve persistent inconsistencies across both research fields but also offers a roadmap for translating ecological concepts into scalable, field-ready solutions that treat context-dependency as a core design parameter. Further empirical validation remains essential to strengthen these linkages and refine predictive frameworks for apparency-based management.

## Data Availability

No new data were created or analyzed in this study. Data sharing is not applicable to this article.
